# The “Target Sign” in a 46-Year-Old Patient with COVID-19 Pneumonia

**DOI:** 10.1155/2021/9956927

**Published:** 2021-10-29

**Authors:** Alexandra Pérez Pérez, Rahul Lazarus, Anju Dubey

**Affiliations:** ^1^Department of Radiology, SUNY Downstate Health Sciences University, 450 Clarkson Ave, MSC #1198, Brooklyn, NY 11203, USA; ^2^SUNY Downstate Medical Center/Kings County Hospital, USA

## Abstract

COVID-19 has various imaging manifestations, most commonly peripheral ground-glass opacities with a basilar posterior predominance. Less common imaging manifestations include consolidations, findings typical of organizing pneumonia, such as “halo” or a “reverse halo” sign, and vascular enlargement. Our case describes a “target sign” on CT, which is uncommon but is increasingly being recognized. The target sign consists of a central nodular opacity with surrounding ground-glass opacity, then a surrounding relatively lucent ring, and a more peripheral ring of consolidation or ground-glass opacification. This may be the sequela of focal vascular enlargement, endothelial injury, microangiopathy, and perivascular inflammation. The case described involves a 46-year-old male who presented with subjective fevers, nonproductive cough, and hypoxia, subsequently diagnosed with COVID-19. CT imaging performed as part of initial work-up revealed multifocal ground-glass opacities scattered throughout the lung parenchyma, as well as multiple target sign lesions. Although it is a rare finding, the target sign, when present, may suggest the diagnosis of COVID-19.

## 1. Introduction

COVID-19, a disease caused by the severe acute respiratory syndrome coronavirus 2 (SARS-CoV-2), can have variable presentations on CT imaging of the chest. Though CT is not routinely recommended for screening and diagnosis, it may be a useful tool when diagnostic tests are not available or to assess complications. Imaging findings may be nonspecific, although common findings include bilateral, peripheral, multifocal, and ground-glass airspace opacities, usually with basilar predominance [1]. Other nonspecific imaging findings, such as interlobular septal thickening and consolidation, have been seen with progressive infection. Less common manifestations include a halo sign and reverse halo sign [1]. The halo sign is described as a central dense nodular opacification surrounded by a ring of ground glass and is associated with hemorrhage as seen in fungal infections, as well as other etiologies such as malignancies and cryptogenic organizing pneumonia. The reverse halo sign, which appears as a peripheral ring of consolidation surrounding a central focus of ground-glass opacity, was originally described with cryptogenic organizing pneumonia but has also been described with fungal infections, malignancies, and other etiologies [2].

The case presented describes another atypical imaging manifestation on CT, the “target sign,” which we define as a nodular central opacity with surrounding ground-glass opacity, surrounded by a ring of relative lucency and another ring of ground-glass opacity or consolidation. This finding may represent infection-mediated pulmonary vascular disease characterized by endothelial injury, microangiopathy, thrombosis, and perivascular inflammation [3, 4].

## 2. Case Report

A 46 year-old male with past medical history of hypertension, diabetes mellitus, and chronic alcoholism presented to the emergency department with symptoms of nausea and vomiting that had started 10 days prior to arrival. The patient also reported associated subjective fever, chills, diarrhea, fatigue, nonproductive cough, and weight loss, which he attributed to poor appetite and decreased oral intake. The patient stated that he had COVID-19 exposure through family members who lived in the household.

On physical examination, the patient was diaphoretic, ill-appearing, and dehydrated, with tachycardia of 117 bpm and a respiratory rate of 20 breaths per minute. The patient was afebrile at time of evaluation, and all other vital signs were normal. The remainder of the physical examination was unremarkable.

**Figure 1:**
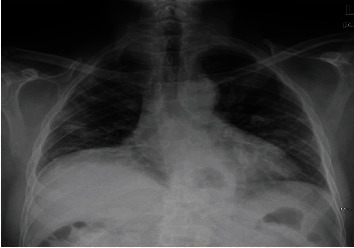
AP portable radiograph showing low lung volumes and patchy linear opacities in the periphery of both lungs.

Shortly after arrival, however, the patient developed a fever of 102.6°F and oxygen saturation decreased to 84%. Initial chest radiograph showed patchy peripheral opacities predominantly in the bilateral midlung fields (Figure 1).

Real time polymerase chain reaction (RT-PCR) test was positive for COVID-19. The patient was admitted for acute respiratory failure. Solumedrol and dexamethasone were administered, and patients' hypoxia improved. The patient was also started on ceftriaxone and azithromycin daily for bacterial coverage.

**Figure 2:**
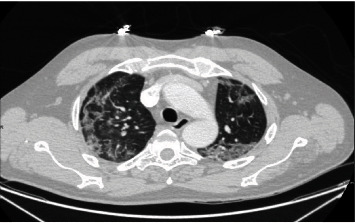
Axial contrast-enhanced chest CT in lung window showing bilateral peripheral ground-glass opacities.

**Figure 3:**
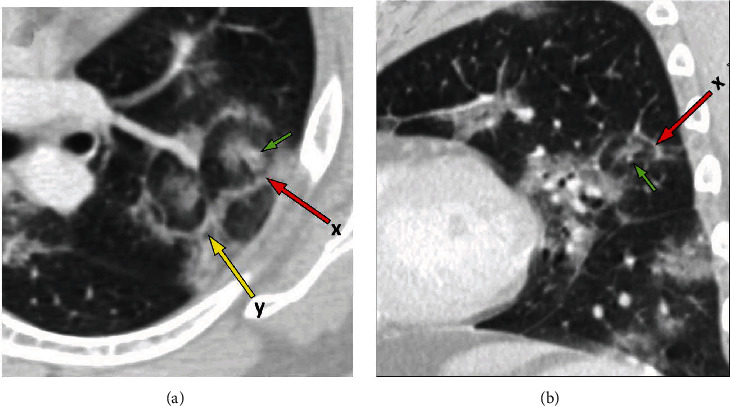
(a) Obliqued axial image demonstrating two adjacent target lesions, labelled “x” marked by the red arrow, and “y” marked by the yellow arrow. Lesion “a” clearly demonstrates a “central dot” (small green arrow) which corresponds to a pulmonary artery branch. (b) Oblique sagittal image through lesion “x” (red arrow) depicting the “central dot” or small pulmonary artery branch (small green arrow).

**Figure 4:**
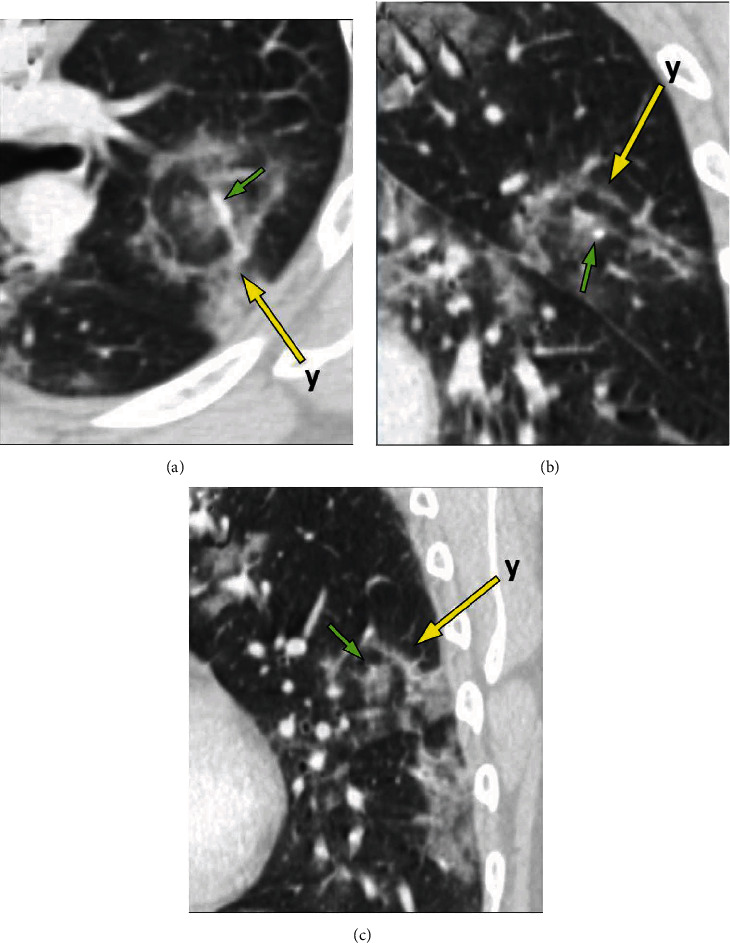
(a) Axial image at a level superior to Figure 3(a) depicting target lesion “y” (yellow arrow). The small green arrow shows the prominent central vessel. Oblique coronal (b) and sagittal (c) images through lesion “y” (yellow arrow), also showing prominent central vessels (small green arrows).

Chest CT revealed bilateral, multifocal, and peripheral ground-glass opacities consistent with known COVID-19 infection (Figure 2). In addition, two “target” lesions were also identified demonstrating nodular central opacities surrounded by a ground-glass halo, then a ring of relative lucency which in turn was surrounded by a ring of peripheral ground-glass opacity or consolidation (Figures 3 and 4).

Empiric antibiotic therapy was discontinued as findings were compatible with COVID-19 pneumonia without superimposed bacterial infection, and initiation of a 5-day course remdesivir therapy with continuation of dexamethasone was recommended by the Infectious Diseases consult service.

The patient's oxygen requirement increased and was transitioned to 10 L via high flow nasal cannula. The patient completed the course of remdesivir and dexamethasone, but as he was still desaturating upon ambulation, he was continued on high flow oxygen and extended course of dexamethasone for a total of 10 days.

The patient remained in the hospital for several days until improvement of respiratory status and successful weaning of oxygen to 3 L via nasal cannula. The patient was eventually discharged home with arrangements for home oxygen therapy, which was ultimately discontinued as an outpatient.

## 3. Discussion

COVID-19 viral pneumonia is associated with a myriad of imaging findings, some of which have been shown to vary depending on the disease stage, although the more common findings, namely, bilateral lung involvement and ground-glass opacities predominantly in a peripheral distribution, may present at any stage [1].

Our case presented scattered areas with a central nodular density surrounded by a ground-glass halo, then a ring of relative lucency with an adjacent ring of consolidation or ground-glass opacity, resembling a “bullseye” or “target.” There have been a few reports of similar findings [5, 6]. Additional cases have shown slightly different findings, such as a “Ring of Fire” appearance [7] or “Rings of Saturn” [8] or “Double Halo” appearance [9]. Some studies have speculated that the target sign appearance may be due to similar pathophysiological processes found in organizing pneumonia [5, 6]. Organizing pneumonias may present as a reverse halo sign, which is thought to be due to granulation tissue polyps within alveolar ducts with occlusion of the broncho-vascular bundle, with a peripheral ring of dense consolidation correlating to inflammatory and infectious debris [10]. This mechanism, however, would not account for the central dense consolidation seen in the target sign. A possible explanation may include the pathophysiology of the halo sign, in which the central dense nodular opacity reflects an area of central necrosis or hemorrhage, surrounded by inflammatory changes in the adjacent lung parenchyma. In our case, the central nodular opacity was generally next to a pulmonary arterial branch (see “central dots” in Figures 3 and 4). It is well known that the ACE2 receptor, which the SARS-CoV-2 virus uses for entrance into cells, is found in many organs, including blood vessels [1]. Multiple studies have found vascular and perfusion abnormalities in COVID-19 [3, 4]. A commonly encountered finding in COVID-19 pneumonitis is that of vascular enlargement [1, 4, 6, 11]. Normally, the lung's response to pneumonia is induction of pulmonary vasoconstriction, which leads to blood shunting away from the affected parenchyma towards less affected regions, producing a beneficial physiological ventilation-perfusion match [3]. It has been inferred that COVID-19 disrupts the lung's physiologic vasoregulation, which could explain why some severe hypoxemic cases have shown little or no parenchymal findings. Lung autopsies in deceased COVID-19 patients show new vessel angiogenesis, small and medium-vessel vascular enlargement with extensive endothelial injury, and endothelialitis, as well as widespread thrombosis with microangiopathy and angiocentric inflammation [1, 3, 4, 6].

Therefore, the target sign encountered in our case may be attributed to COVID-19's vascular predilection, leading to focal enlargement of the pulmonary artery within the bronchovascular bundle, with adjacent hemorrhage or edema, with the peripheral ring of opacity possibly due to organizing pneumonia.

Limitations in our case are the lack of direct pathologic correlation. COVID-19 infection was confirmed using standard RT-PCR. After treatment and resolution, the patient was discharged home, and no follow-up imaging was available to assess progression or resolution of target lesions and vascular findings. Another important consideration is the lack of optimal assessment of the pulmonary arteries, as the available imaging was not performed as an angiography protocol. Multiple factors may have contributed to the imaging findings in this case and assessment of whether findings are predominantly vascular necessitate more similar cases and further investigation with pathologic correlation. Nevertheless, the radiologic appearance of the target sign may have utility in suggesting the possibility of COVID-19 infection when the diagnosis is not known.
